# Boolean Modeling of Biological Network Applied to Protein–Protein Interaction Network of Autism Patients

**DOI:** 10.3390/biology13080606

**Published:** 2024-08-10

**Authors:** Leena Nezamuldeen, Mohsin Saleet Jafri

**Affiliations:** 1 School of Systems Biology, George Mason University, Fairfax, VA 22030, USA; lnnezamuldeen@kau.edu.sa; 2 King Fahd Medical Research Centre, King Abdulaziz University, Jeddah 21589, Saudi Arabia; 3 Center for Biomedical Engineering and Technology, University of Maryland School of Medicine, Baltimore, MD 21201, USA

**Keywords:** ASD, Boolean modeling, autism, mTOR, Wnt, autism therapy

## Abstract

**Simple Summary:**

Boolean modeling is a graphical analytic approach used for analyzing qualitative models of biological systems, including protein–protein interaction networks. This study performed an analysis of the protein–protein interaction network related to four individuals diagnosed with Autism Spectrum Disorder (ASD) to identify the underlying etiology of the observed phenotype. The genetic mutations in each of these patients have been found to be convergent with the widely recognized Wnt and mTOR signaling pathways, which have previously been implicated in the development of ASD. The disturbance in the activity of these genetic mutations caused abnormal activation levels of critical proteins such as β-catenin, MTORC1, RPS6, eIF4E, Cadherin, and SMAD, which regulate gene expression, translation, cell adhesion, shape, and migration. The varied functions of these proteins contribute to the observed traits in these individuals, yet they reveal potential therapeutic options for them.

**Abstract:**

Cellular molecules interact with one another in a structured manner, defining a regulatory network topology that describes cellular mechanisms. Genetic mutations alter these networks’ pathways, generating complex disorders such as autism spectrum disorder (ASD). Boolean models have assisted in understanding biological system dynamics since Kauffman’s 1969 discovery, and various analytical tools for regulatory networks have been developed. This study examined the protein–protein interaction network created in our previous publication of four ASD patients using the SPIDDOR R package, a Boolean model-based method. The aim is to examine how patients’ genetic variations in INTS6L, USP9X, RSK4, FGF5, FLNA, SUMF1, and IDS affect mTOR and Wnt cell signaling convergence. The Boolean network analysis revealed abnormal activation levels of essential proteins such as β-catenin, MTORC1, RPS6, eIF4E, Cadherin, and SMAD. These proteins affect gene expression, translation, cell adhesion, shape, and migration. Patients 1 and 2 showed consistent patterns of increased β-catenin activity and decreased MTORC1, RPS6, and eIF4E activity. However, patient 2 had an independent decrease in Cadherin and SMAD activity due to the FLNA mutation. Patients 3 and 4 have an abnormal activation of the mTOR pathway, which includes the MTORC1, RPS6, and eIF4E genes. The shared mTOR pathway behavior in these patients is explained by a shared mutation in two closely related proteins (SUMF1 and IDS). Diverse activities in β-catenin, MTORC1, RPS6, eIF4E, Cadherin, and SMAD contributed to the reported phenotype in these individuals. Furthermore, it unveiled the potential therapeutic options that could be suggested to these individuals.

## 1. Introduction

Multiple genetic variants impact neuropsychiatric conditions to generate a wide range of phenotypic effects. The prospective interactions of these mutations can be complex, having an impact on multiple factors such as neurotransmitter function, neural development, and synaptic plasticity. The cumulative impact has the potential to increase susceptibility to several conditions, including schizophrenia, intellectual disability, and autism spectrum disorders. Understanding these connections is of the utmost importance in understanding the complex genetic landscape of neuropsychiatric disorders [1,2]. 

Several workflows and software tools have been developed for the purpose of extracting genetic and variant information. These include GATK4 [3] from the Broad Institute, VCFtools v4.2 [4], Pathway Studio v7.0.from ELSEVIER, DeepVariant v1.6.1 [5], Free Bays v1 [6], and SAMTools v1.20 [7]. However, the process of interpreting the relation between genetic variants and the etiology of disorders requires the active participation of human experts and the manual curation of scientific literature. Moreover, de novo mutations identified in complex neuropsychiatric disorders, such as autism, schizophrenia, and intellectual disability, which were detected through family-based whole genome or whole-exome sequencing studies but not through genome-wide association studies (GWAS), are another challenge in interpreting their relation to disease etiology. The limited availability of fundamental resources within genomic and proteomic databases related to de novo mutations makes the prediction of the impact of these mutations and their significance in relation to the causation of the respective diseases difficult [8,9,10,11]. An additional challenge emerges from the insufficient utilization of paralogous proteins, protein families, and protein domains in the interpretation of genetic variants and their correlation with the disease phenotype in genome-wide association studies (GWAS) related to neurological diseases. This limitation hinders the comprehension of the mutations detected in newly identified genes. These genes may have extensively researched paralogs, or they may be affiliated with protein families or exhibit a conserved domain. This would enable researchers to determine the impact of these mutations, particularly if the level of conservation is significant among the proteins, their paralogs, protein domains, or protein families [12,13]. 

The process of identifying and interpreting genetic variants linked to disease causation requires a structured approach to ensure the relevance of the findings [2,14]. This systematic approach should include a range of investigations, including identifying the function associated with the genetic variant site, finding the other proteins that interact with the protein with genetic variant, and discovering the biological pathway to which this gene belongs. Some studies used this organized approach and included supportive evidence from protein–protein interaction studies and animal models [11]. While there is potential to generate well-structured systems, it is necessary to model these systems in order to gain a deeper understanding of the biological mechanisms underlying the impact of genetic variations on disease development. Biological cells contain proteins and other molecules that interact in a coordinated manner to facilitate biological activities and maintain cell function. Cellular functions are defined by regulatory networks that represent molecular interactions. Regulatory and protein–protein interaction networks are often regarded as qualitative models unless they have been thoroughly studied and include kinetic data [15].

Boolean network models are a type of graphical qualitative model that was initially presented by Kauffman in 1969. These models have been widely used in multiple previous studies to investigate biological regulation networks [16,17,18,19]. The application of graph dynamical system methods is considered to be one of the most direct approaches to understanding the behavior of biological regulatory networks. These approaches do not depend on quantitative data, such as concentration levels and reaction rates, which are essential for modeling kinetic systems [20]. In the framework of Boolean networks, genes are represented as binary variables, which typically indicate a state of gene activity (active or inactive, 1 or 0). The network is characterized by logical rules that define the process in which the state of each gene is regulated by the states of its regulatory genes [15]. A discrete time series of Boolean systems, whether with a synchronous or asynchronous update, can be characterized as follows: the state of the entire system at time t + 1 is determined by the state of the network at time t through the evaluation of logical functions among the nodes. In this study, the SPIDDOR (Systems Pharmacology for Efficient Drug Development on R) R package, available in https://github.com/SPIDDOR/SPIDDOR (accessed on 29 June 2024), a tool specifically designed for Boolean network modeling, was used [21]. The aim is to investigate the effect of mutant genes, previously found in individuals with autism in [22], on cellular processes and their association with the appearance of the autism phenotype. Based on a comprehensive review of previous research, it is evident that these mutant genes have an important impact on the functioning of the Wnt and mTOR signaling pathways, as well as their convergence. A protein–protein interaction network was constructed and studied by utilizing mutated genes and other molecules within the Wnt and mTOR pathways. The analysis revealed changes in certain protein activity levels, which explain the appearance of the phenotype. Moreover, a proposal was made to predict precise treatments for these patients, given the behavior of the network molecules.

## 2. Materials and Methods

### 2.1. The Clinical Applications and Description of the Biochemical Pathway

Four patients with ASD were discussed in [22] with a confirmation of the genetic variations’ role in ASD (Table 1). The selection of these patients to be included in this study was based on the fact that the genes with variants in these patients converge in the mTOR and Wnt signaling pathways, which are considered to be the core pathways implicated in autism spectral disorder. These pathways are well known to be affected in autism patients, as described in many previous papers that suggest targeting these pathways for autism treatment. However, two proteins, SMS and NGF, were removed from the analysis because of their lack of convergence with the two signaling pathways. Patients 1, 2, 3, and 4 are substituted for patients 64, 73, 37, and 43 in [22], respectively. In summary, the inclusion/exclusion criteria are as follows:

1. All the patients that only have polymorphism mutations (mutations that are common in the population) are excluded.
2. Any genes that are sex-related with regards to cellular metabolism, such as SMS and SEMG2, are excluded.
3. Any genes that are not involved in Wnt or mTOR pathways are excluded

By adopting this strategy, we have the comparison on a tractable model so that the Boolean network model can give a clear understanding of signaling changes caused by the set of mutations in each patient.

There is an association between the genes carrying the mutations and autism spectrum disorder. First, the clinical study by Mubarak et al. [22] cited in the manuscript found that the genes INTS6L/USP9X/RPS6KA6, IDS, FLNA, and SUMF1 are associated with Autism in the patients participating in their study. Second, Both IDS and SUMF1 have been associated with patients with syndromic autism. Syndromic autism refers to instances of autism spectrum disorder that are linked to a more extensive medical disease, typically a syndrome, such as multiple sulfates deficiency. Autism cases that do not have any link with other conditions, which account for the majority of all autism cases, are referred to as non-syndromic autism.

#### 2.1.1. Mutation in SUMF1

Multiple Sulfatase Deficiency (MSD) and its progressive nature, in part due to the accumulation of glycosaminoglycans (GAGs). One of the clinical features of this deficiency is Autism [23]. Several studies have stated that multiple sulfatase deficiency is caused by a mutation in SUMF1 and that autism features appear in these patients [24,25,26,27]. Hijazi et al. have observed that autistic features were present in 3 of 6 (50%) patients in their study. Sheth et al. noted that autism spectrum disorder is likely to be a secondary manifestation arising due to multiple sulfatase deficiency and is often misdiagnosed as autism.

#### 2.1.2. Mutation in IDS

Mucopolysaccharidosis II (MPS II), also known as Hunter syndrome, is a rare, X-linked disorder caused by a deficiency of the lysosomal enzyme iduronate-2-sulfatase (IDS), which catalyzes a step in the catabolism of glycosaminoglycans (GAG) (Heparan sulfate (HS)). In patients with mucopolysaccharidosis II, glycosaminoglycans (GAG) accumulate within tissues and organs, contributing to the signs and symptoms of the disease [27]. This paper found autism symptoms in a child with Hunter syndrome [28]. Another paper stated the following: Autistic behaviors are so pervasive in MPS-III that they lead to a misdiagnosis of autism spectrum disorder (ASD). They also manifest in other forms of mucopolysaccharidosis (MPS) characterized by defective metabolism of HS, such as MPS-II and MPS-I, caused by disruptions to the enzymes iduronate-2-sulfatase and α-L-iduronidase, respectively, which leads to accumulation of HS and other GAGs [29]. 

#### 2.1.3. INTS6L/USP9X/RPS6KA6 and FLNA

The genes INTS6L/USP9X/RPS6KA6 and FLNA are non-syndromic autism. Duplication of Chromosome X, which contains the genes INTS6L/USP9X/RPS6KA6 and FLNA, is associated with Autism and Schizophrenia in ClinVar [30] (https://www.ncbi.nlm.nih.gov/clinvar/variation/488014/?oq=488014&m=Single+allele, accessed on 1 August 2024). Studying the distinctions and resemblances, such as shared pathways, between syndromic and non-syndromic cases might offer valuable understanding into the underlying mechanisms of autism and open up promises for developing novel therapeutics for autism.

To understand and investigate the effect of these genetic variants on the development of the disease, a comprehensive manual annotation in protein databases and previous literature was made, and we found interactions between the proteins listed in Table 1 and other molecules within the Wnt and mTOR pathways, which revealed a convergence between these two signaling pathways. The manual annotating included multiple steps. First, the identification of the specific sites of genetic variants within the protein structure. Second, an analysis was conducted to assess the functional characteristics of the genetic variation locations. Finally, an extensive review and evaluation of previous research related to the proteins listed in Table 1 was made. The proteins’ paralogs and families were considered too. The primary databases used to search for information about these proteins were UniProt, the conserved domain database (CDD), PROSITE, iPTMnet, and PubMed. An example of this manual curation, reviewing the INTS6L profile in UniProt and conserved domain databases, revealed that the protein has a von Willebrand factor type A (VWFA) domain at the N-terminus, a DEAD-box motif, and a functioning C-terminal domain. The genetic variant of INTS6L is not located in any functional regions of the protein. Moreover, the INTS6L has not been well investigated previously. However, its paralog, INTS6, has been thoroughly examined. Previous research has demonstrated that INTS6 plays a crucial role in regulating the inhibitory growth of dorsal cells via the Wnt pathway [31]. A similar manual review was made for the other proteins listed in Table 1 in order to determine whether their genetic variants are located in functionally important regions of the proteins. 

### 2.2. Boolean Modeling of the PPI Network

After discovering from the manual annotations, the relations between the proteins in Table 1 and the other molecules in the Wnt and mTOR pathways, Boolean algebra, was used to convert these relations and turn it to a Boolean model.

The conjunction (AND), represented by (&); the disjunction (OR), represented by (|); and the negation (NOT), represented by (!), are the primary operators of Boolean algebra. Biological processes are non-linear, and certain genes may require extended activation periods for their regulator genes to become active. For example, it is typical to find statements in literature that say gene A increases or decreases the expression of gene B. This statement suggests that gene A can either enhance or suppress the activity of gene B, depending on whether gene B is affected by other signals that increase or decrease its activation. For this reason, the developers of SPIDDOR incorporated additional modulator operators to enhance the precision of defining various biological activities. The modulator operators define the activation and deactivation of Boolean functions in a network-based model. They are useful in simulating and analyzing complex systems. Simply put, these operators are used when there is no direct interaction between the genes, such as in the example of gene A increasing the expression of gene B. Also, these operators generate the activation of gene A only based on the assessment of the activation of its regulator nodes in the last n iterations of the simulation, with a default value of n = 3. This implies that the simulation will evaluate the activation of the regulators of gene A in the three previous iterations and then determine whether gene A is activated or inhibited. 

The first operator is the threshold operator, represented as THR_(GENENAME) [n]. The threshold operator compares a vector of values to a certain set of values that partitions the multidimensional space with a hyper plane to classify the vector as false or true. In the context of the model, if the regulator (upstream protein) has been activated within the last n iterations (default value of n = 3 in the model), the target protein will be considered to be active. For example, GENEB = THR_GENEA [3] indicates that the Boolean variable GENEB will be activated if its regulator (GENEA) has been active in the last 3 iterations of the network simulation. The second operator is the modulator operator denoted by MOD_(GENENAME) [n]. This operator functions similarly to the THR operator but exclusively affects nodes that have modulation interactions within the Boolean functions of the network. For example, GENEB = MOD_GENEC [4] & MOD_GENEA [4]. This indicates that the activation of GENEB depends on the modulation status of GENEC and GENEA in the last 4 iterations of the network simulation. The third operator is the ANY operator, denoted as ANY_GENENAME) [4]. The ANY operator determines whether a protein is activated or inactivated (Boolean functions true or false) in any of the last n iterations based on the conditions defined by the thresholds. For example, GENEB = GENEA & ANY_GENEC [4]. This expression signifies that the activation of GENEB depends on the presence of GENEA, as well as certain conditions involving ANY_GENEC [4] evaluated over the last 4 iterations.

### 2.3. Boolean Network Simulation

By default, the initial state for all nodes is set to 0. The network’s dynamic trajectory is computed by utilizing the SPIDDOR library in R. This library has a dynamic evolution function that simulates the network in an asynchronous mode by default. This simulation function updates the states of the nodes based on the Boolean operator assigned to each node to activate or deactivate it in every iteration. Boolean models rely on two updating methods: synchronous and asynchronous. In the synchronous updating method, the network’s node state at each time step depends on the node state from the previous iteration. This would lead to a predictable trajectory of the network. In other words, this would lead to the network’s nodes continuously achieving the same state after a certain number of iterations. This is unrealistic because of the absence of chronological synchronization in cellular processes. For this reason, the asynchronous mode was chosen rather than the synchronous mode because the regulation of biological networks is not characterized by a linear sequential trajectory. Moreover, the authors of [21] suggest that the biological regulatory networks and their coordination in timescales are realistically represented when using the asynchronous random method rather than the synchronous method. The random asynchronous method updates the network’s node state depending on the most recent regulator node updates, either from the previous or current iteration. Node state updates are random in each iteration, introducing heterogeneity into the model. As a result, this would present a variability in the model and different final states, which would nearly characterize the regulation networks in biology.

The simulation was iterated 2500 times over an average of 100 time steps. The resulting output consists of 2500 matrices that describe the statuses of the nodes at each time step, indicating whether they are activated or inhibited (1 or 0). Afterward, the average simulation function inside the SPIDDOR library in R is used to reduce the stochasticity of the 2500 output matrices. In each time step, for each node, the average simulation function uses the Wald method to determine a 95% confidence interval for the fraction of successes (activation, 1) [32]. The Wald method (Equation (1)), a commonly applied method, is used for calculating binomial confidence interval in discrete distribution of binomially observation experiments (activation/inhibition) in our regulatory network

(1) $$p\pm z\sqrt{p\left(1-p\right)}/n$$

where *p* is the probability that the node is activated at time step t over the n independent realizations of the stochastic process, *n* is the total number of simulations, *z* is the quantile of 1 − α/2 (*z* = 1 − α/2) of a standard normal distribution, and α is the error rate. The error at a 95% confidence level is 0.05, with a corresponding *z*-value of 1.96.

In order to assess the effect of genetic variants in the network, the SPIDDOR library in R allows for the modification of the activity levels of nodes to mimic a mutation-like effect. For example, if a mutation-like effect was appointed to a node to be 30% active (that would be the activation of the node during the simulation for example), the activation of the node will be 30% of the times and does not always activate when its regulator nodes activate. The proteins’ activity levels in the network that has a genetic mutation (as indicated in Table 1) were altered to 50% and 0%. The 50% alteration refers to the possible loss of protein function, such as the total loss of one allele. On the other hand, the 0% alteration indicates the entire loss of protein function. The average simulation function is used again to examine the impact of genetic variants on protein function, including partial or complete loss of function.

### 2.4. Attractor Analysis

The Boolean models have a unique feature of having a state that is in between two binary states, namely 1 and 0. Therefore, the system’s dynamic trajectory tends to revisit previous states and eventually is trapped in one of them during the simulation process. The states in which the system trajectory becomes trapped are commonly referred to as steady states or attractors. The observed oscillating behavior in the system is attributed to the presence of a feedback loop. The attractors fall into two categories: (i) Point attractor that has a single state. In this attractor, there is a single state for the system to return to after transitioning between many states. (ii) Cyclic attractors are composed of many steady states. If a system oscillates cyclically, this is referred to as a simple cyclic attractor. The unique feature of the synchronous method is that each state has only one possible successor state. When the system exhibits irregular oscillations in which they follow close to the same limit cycle but not exactly the same, it is commonly referred to as a complex attractor. On the other hand, with the asynchronous method, the stochastic or discrete switching between states causes the model to switch between attractors in what is known as stochastic switching. As a result, the system does not oscillate in cycles when this method is used since each state has an abundance of alternative successor states. The SPIDDOR library in R includes a function called “get attractor” that is utilized in asynchronous mode to determine the steady state of the nodes in the network. The asynchronous mode is chosen again because, as mentioned earlier, it will represent the realistic nature of the biological regulation network processes. In networks, the use of an asynchronous updating method usually results in the convergence into stochastic switching between attractors. For better results, the get attractor function may calculate the likelihood that a specific node is ON or 1 inside the attractor and visualize these probabilities using bar graphs.

### 2.5. Sensitivity Analysis and Clustering

The evaluation of the network’s sensitivity to molecules’ perturbations is important in order to evaluate the substantial impact of nodes on the dynamics of the system. In the context of Boolean models, the sensitivity test involves the computation of a ratio of the probability of a single node being activated in a perturbed state of the network and the activation of the same node in the network’s normal state. Perturbed conditions refer to two scenarios inside the system: the overexpression of particular nodes leading to their sustained activation throughout the simulation or the loss of individual nodes resulting in their sustained deactivation during the simulation. The knockout matrix function in the SPIDDOR package in R was used to measure the sensitivity of our regulation network. The knockout matrix function was used instead of the overexpression matrix function. The careful curation of past research on proteins with genetic variants suggests these mutations primarily cause a decrease or knockout effect on the proteins’ function rather than causing an overexpression of the proteins. The knockout matrix function is designed to perform synchronous-asynchronous network simulation. In synchronous mode, the network simulation finds the attractor and calculates the probability of the other node’s activation in the normal state of the network. Each node in the network is knocked out in asynchronous mode, and the network simulation calculates the attractor and probability of other node’s activation within the network. The resulting square matrix generated by this function provides clarification on the impact of knocking out the column node on each row node. Each cell in the matrix represents the ratio of a node’s activation in its normal state to its perturbed state resulting from the knockdown of a column node. The developers of the R library [21] referred to the ratios in each of the matrix cells as the Perturbation Index (PI), and they defined the equation used to calculate each matrix cell’s value as follows in Equation (2), “for a given node i under a perturbation in j” [21]

PI_J_i_ = Probability(i)_Perturbation(j)_/Probability(i)_Normal_ (2)

To enhance the visual representation of the output matrix, the developers utilize a heatmap method. They assign a numerical range of (−1, 1) to convert the ratios into numerical values. Furthermore, they propose expanding the range of values if the network being modeled is more complicated, as indicated in Table 2. The negative values indicate a reduced level of activation in the nodes represented by blue colors, contrasting with the positive levels denoted by orange colors. The gray color is used to represent a 0-value, indicating that there are no significant differences between the perturbed and normal conditions. The default sensitivity parameter is preconfigured to 20%, indicating that the heatmaps generated by the heatmap function will be colored only when there is a variation in the attractor states above 20%.

Moreover, the developers of the R library have incorporated a hierarchical clustering method into the sensitivity analysis. This enables the grouping of nodes with similar impacts on the system’s nodes when subjected to knockout or overexpression. The clustering is based on the distance between nodes, and dendrograms are included in both the PI’s matrix and the heatmap. The Euclidean matrix is used as the method for quantifying the distance between the nodes’ PI. Furthermore, the average-linkage strategy is utilized to merge the clusters. The mathematical formula used to quantify the spatial separation between a knockout event occurring in node A and a knockout event occurring in node B is expressed as follows in Equation (3):

(3) $$d\;(A,B)=\sqrt{\sum_{i=1}^{n}(PI\_B_{i}-PI\_A_{i}{)}^{2}}$$

where *n* is the number of nodes in the network, and *PI_A* and *PI_B* are the perturbation indexes of the nodes under the knockout in A and B.

## 3. Results

### 3.1. Variants Annotation

**Figure 1:**
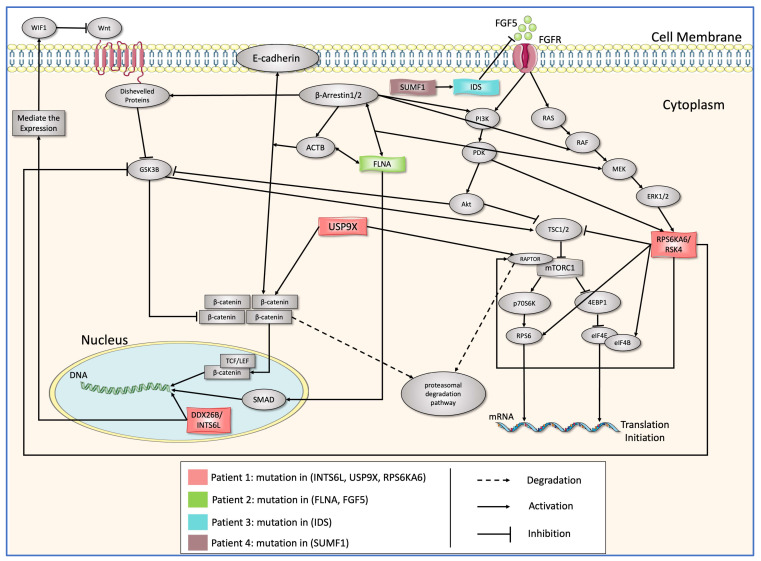
Schematic representation of the mutated proteins in four patients colored with red, green, cyan, and brown with arrangements of their roles on Wnt and mTOR signaling pathways [33].

After annotating and reviewing the biological databases and publications on the proteins (Table 1) reported to cause ASD and after discovering their functional effect on the biological pathways, we found that these proteins activate, inhibit, and mediate molecule expression in the two important signaling pathways Wnt and mTOR. The protein–protein interaction network (Figure 1) explains protein relationships.

The analysis of the protein–protein interactions illustrated in Figure 1 revealed key outcomes. INTS6, which is a paralog of INTS6L, imposes a major impact on the Wnt pathway. Furthermore, it was observed that mutations in INTS6 are associated with dorsal cell proliferation. The expression of WIF-1, the inhibitor of Wnt protein, is upregulated by INTS6, hence modulating the activity of the Wnt pathway [31,34,35]. USP9X interacts physically with RAPTOR and β-catenin in order to prevent their degrading by the proteasomal degradation pathway [36,37,38]. RPS6KA6/RSK4 has high expression levels in the brain, and its mutation has been suspected of non-specific intellectual disabilities and developmental delays [39,40]. The RSK enzymes catalyze the phosphorylation of RAPTOR, resulting in the activation of the mTOR pathway [41,42,43]. Moreover, they inhibit the enzymatic activity of GSK3β, resulting in the accumulation of β-catenin and subsequent cell survival [44]. The binding of FGF5 to the FGFR1 receptor initiates the activation of many signaling pathways, such as PI3K/Akt, RAS/MAPKs, and PLCγ/DAG. These pathways are involved in a diverse array of cellular proliferative processes, including embryonic development, cellular growth and survival, and the repair of tissues [45]. The modulation of FGF signalling has been implicated in various pathological conditions, including skeletal disorders, malignancies, dwarfism, alopecia, neural plasticity, and autism [46,47,48]. The FLNA-actin interaction is of crucial significance in cellular adhesion. The C-terminal region of the protein contains a total of 24 repetitive sequences, which play a significant role in mediating protein–protein interactions. Mutations in the FLNA gene have been found to disrupt critical physiological mechanisms and have been associated with various medical conditions, such as autism [49,50]. The mutation in the FLNA gene resides inside repeat 22, where the protein interacts with β-arrestin to initiate the activation of MEK. Additionally, this contact facilitates the translocation of SMAD to the nucleus, where it carried out the transcription of specific genes [51,52,53]. IDS is an essential lysosomal sulfatase involved in protein metabolism, more specifically in the degradation of glycosaminoglycans from its substrates dermatan sulphate and heparan sulphate [54]. Heparan sulphate is a crucial and specific component in the dimerization and binding of FGF/FGFR [55]. As a cofactor, SUMF1 facilitates the metabolism of sulfatase enzymes, including IDS [56]. A mutation in SUMF1 results in an insufficiency of sulfatase enzymes, and it is due to the fact that it is a critical molecule present at the site where the cysteine residue is modified to form formyl glycine residue, which is an essential molecule in the post-translation modification of these enzymes [57]. These relations are translated to Boolean equations, explained, and listed in (Table A1). These equations control Boolean model dynamics.

### 3.2. The Dynamic of the Boolean Simulation

**Figure 2:**
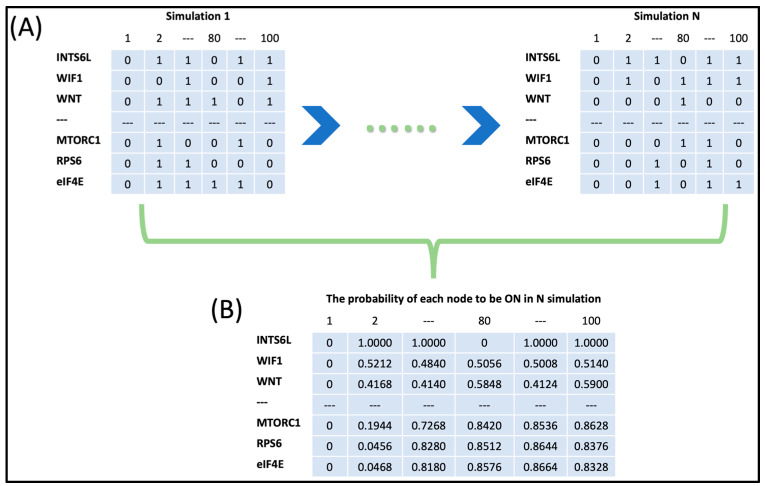
Schematic representation of the algorithm developed in SPIDDOR library in R using the asynchronous method. (**A**) The output of the dynamic evolution function. The rows represent the nodes, and the columns represent the 100 iterations. (**B**) The output of the average simulation function. The probability of each node to be ON calculated from 2500 (N) simulations.

The use of the dynamic evolution function in asynchronous mode showed the trajectory of the molecules in the network according to the relations between them defined by Boolean equations in (Table A1). The output of one simulation for 100 time steps is a matrix encompassing (1 and 0) (Figure 2). The dynamic evolution simulation of 2500 times for 100 steps yields 2500 1 and 0 matrices. In each of the 2500 matrices, rows represent network proteins, and columns indicate the 100 time steps. For example, matrix 1, row number 1 is for INTS6L protein. At time step 1 (column 1), the state of INTS6L is 0, and at time step 2 (column 2), it is 1; this continues until time step 100. The protein INTS6L is activated six times in the first 10 iterations and 60 times in 100 iterations. When a mutation-like effect is introduced to the protein in Table 3, its activity decreases by 50%. The total number of activations of the protein during the whole simulation is reduced to 30 out of 100, which is 50% of its original rate of activation in the normal state (Table 3).

**Figure 3:**
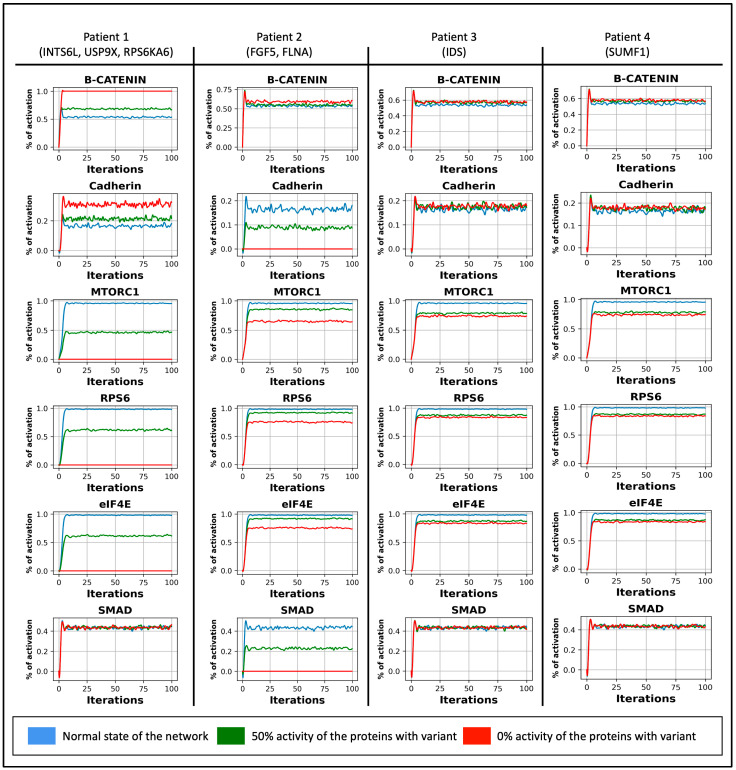
The oscillation of the proteins involved in cell adhesion or DNA transcription and translation in the Boolean system for 2500 simulations in every 100 time steps. The blue color shows when all the proteins are at 100% of their functional effect. The green color when the mutation-like effect was introduced to proteins with variants in each patient to delay their activation by 50%. The red color when the mutation-like effect was introduced to proteins with variants in each patient to knock out their activation.

Two thousand five hundred matrices, such as those in Table 3, are created for all proteins when the dynamic evolution simulation was executed in the network physiological state and when the polymorphism assigned to the proteins with a variant with a rate of 50% and 0%. The average simulation function included in the SPIDDOR R library computed the 95% confidence interval for the proportion of (activation, 1) of each protein in the total of the 2500 matrices in each of the 100 time steps using the 2500 matrices resulted from using the dynamic evolution function. Furthermore, the average simulation function generates a visual depiction of the fluctuations in protein activity within the regulatory network, which helps to illustrate how each protein in the system oscillates in the physiological state as a result of the polymorphism introduced. The average simulation function was performed three times for each of the autistic patients, accounting for the proteins with variants in each patient in the physiological network state when the mutation-like effect is introduced at the rate of 50% and 0% (Figure 3).

**Figure 4:**
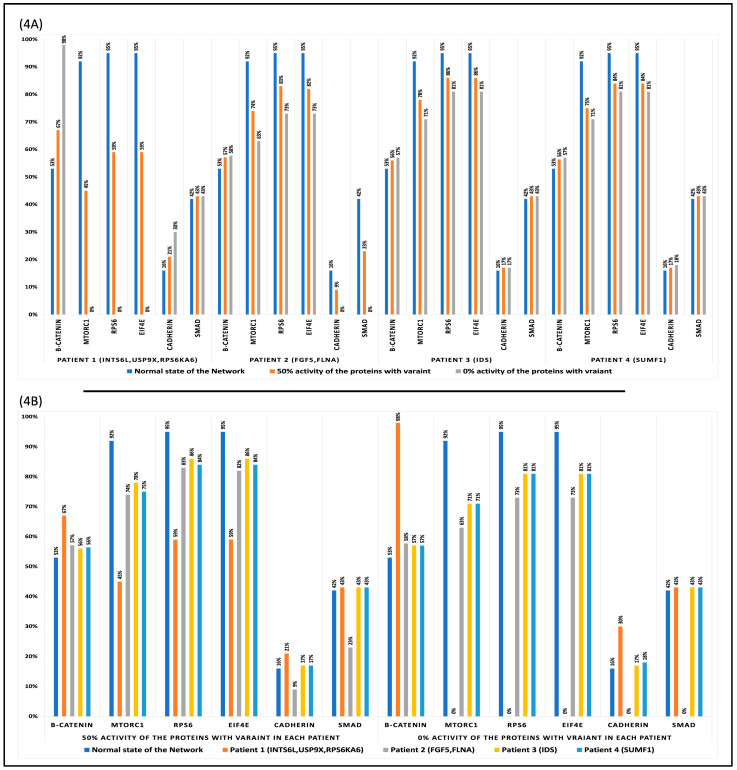
The averages of the datapoints that represent the curves in the Boolean system (Figure 4): (**A**) Showing the oscillation average of each protein in each patient; (**B**) Showing the oscillation average of each protein combined by patients.

The primary actors in cell function are β-catenin, MTORC1, RPS6, eIF4E, cadherin, and SMAD, as they are responsible for maintaining cell adhesion and survival. These proteins showed differences in their oscillations in the regulatory network in the physiological state and when the mutation-like effect was introduced to the proteins with genetic variants at a rate of 50% and 0%. The averages of the datapoints that represent the curves in the Boolean system (Figure 3) were calculated and represented in (Figure 4). In the physiological condition, β-catenin level of activity is 53%, MTORC1 is 92%, RPS6 and eIF4E is 95%, Cadherin is 16%, and SMAD is 42%. When the mutation-like effects were introduced to INTS6L, USP9X, RPS6KA6 in patient1, FGF5, FLNA in patient2, IDS in patient3, and SUMF1 in patient4 at 50% and 0% rate, the simulation results (Figure 4A) revealed a nearly consistent pattern in the activity of β-catenin, MTORC1, RPS6, eIF4E, Cadherin, and SMAD by all patients. The pattern is as follows: increased activity of β-catenin, decreased activity of MTORC1, RPS6, and eIF4E, and ambiguous activity of Cadherin and SMAD. Increased β-catenin activity indicates overactivation of the Wnt pathway in patient 1, but not in patients 2, 3, and 4. In contrast, the mTOR pathway demonstrated a significant reduction in its activity in patient 1 and a significantly lower rate in patients 2, 3, and 4. Cadherin is a protein that is dependent on β-catenin, FLNA, and ACTB. Following cadherin protein binding to β-catenin, α-catenin acts as a facilitator for β-catenin binding to actin filaments.

Cadherin activity increased in tandem with the increase in β-catenin activity in patient 1. Cadherin levels were lowered in patient 2 as a result of the FLNA mutation. SMAD interacts with FLNA and translocates to the nucleus to initiate transcription. SMAD inhibition was observed only in patient 2 in response to the FLNA mutation. Furthermore, the simulation results (Figure 4A) demonstrate a similar pattern observed in patients 3 and 4, where there is no impact on the Wnt pathway but an apparent effect on the MTOR pathway. The simulation results in (Figure 4B) indicated that patients 3 and 4 have significantly lower activations of the mTOR pathway, as stated before, which includes the MTORC1, RPS6, and eIF4E genes. The shared mTOR pathway behavior in patients 3 and 4 is explained by a shared mutation in two closely related proteins.

### 3.3. Attractor Analysis

Finding attractors in Boolean models is frequently associated with the quest for steady states of biological pathways and cellular molecules [15,21,58,59]. When the asynchronous updating method is used, and because of the random nature of the process, an attractor is generated. It is difficult to find steady states in this type of attractor due to the large variety of states that do not oscillate cyclically [60,61]. The steady state of the regulatory network generated in this study is obtained by calculating the probability that a specific node is ON or 1 inside the attractor using the find attractor function of the SPIDDOR package in R. The simulation to find the attractors was repeated 16 times with 1000 time steps in asynchronous mode. This is a summary of the number of states found in the normal condition of the network and in the case of decreasing the activity of the proteins with variants in each patient (Table 4). The numbers are high and explain the randomness of the asynchronous method.

The steady states of the molecules in the network are quite similar to the results obtained by averaging the datapoints of the curves (Figure 4) generated by the average simulation functions in Figure 3. When a mutation-like effect with the two rates of 50% and 0% was introduced to INTS6L, USP9X, and RPS6KA6 in patient 1 and to FLNA and FGF5 in patient 2, the steady state of β-catenin was elevated in patient 1, while the steady state of MTORC1, RPS6, and eIF4E was reduced in patient 1 and 2. The discrepancy in the steady states of cadherin and SMAD proteins among the two patients can be explained by the proteins’ intrinsic properties in terms of protein–protein interactions and their fundamental roles in the cell. Cadherin protein functions in adhesion signaling by binding to β-catenin, which assists cells adhesion and survival. Following Cadherin’s interaction with β-catenin, α-catenin acts as a mediator in the binding of β-catenin to actin filaments, thereby stabilizing cell-cell interactions [62,63]. Cadherin steady state is enhanced in patient 1 when β-catenin activity is increased as a result of INTS6L, USP9X, and RPS6KA6 mutations and dropped in patient 2 as a result of FLNA mutation. Cadherin protein activity requires both β-catenin and FLNA to sustain cell adhesions and connections. SMAD proteins are phosphorylated by TGF-β and bind to FLNA. Then, they translocate to the nucleolus where they interact with transcription factors to trigger the transcription of targeted genes. Except for patient 2, who has an FLNA mutation, patient 1’s SMAD protein activities remain unchanged. The FLNA mutation in patient 2 reduced SMAD activity, and this decrease in SMAD function can be attributed to the discrepancy in transcribing certain genes and cell function, which would support the reason for the appearance of the complex phenotype in patient 2.

Patients 3 and 4 have decreased in the mTOR pathway steady state, which included MTORC1, RPS6, and eIF4E, but not in the steady states of the other proteins. This occurred as a result of the relationship between IDS and SUMF1 in patients 3 and 4, as SUMF1 functions as a co-factor to IDS enzyme.

### 3.4. Perturbation and Hierarchical Clustering Analysis

**Figure 5:**
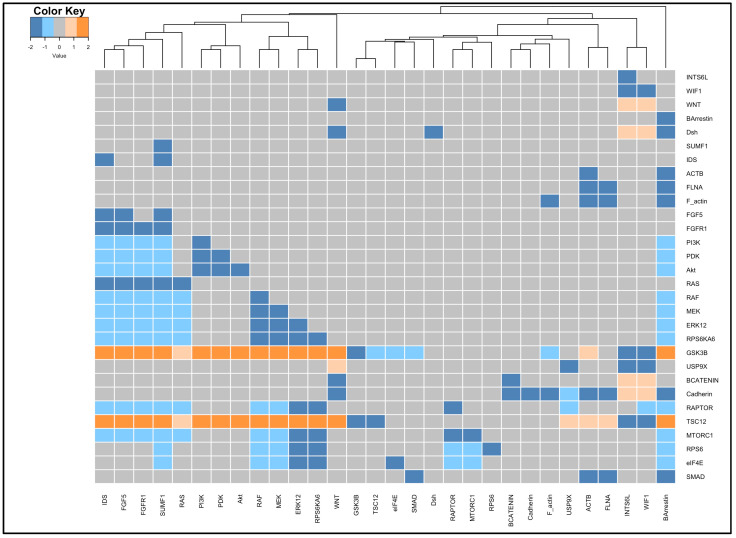
The knockout heatmap representing the perturbation indexes PIs as a result of knocking out each node in the system (columns) and their effect on the other nodes in the network (rows). The heatmap is scaled and colored as follows: PI values close to 1 are taking the value of 0 and colored in gray, PI values between 1.25 and 2 are taking the value of 1 and colored in light orange, PI values greater than 2 are taking the value of 2 and colored in dark orange, PI values between 0.5 and 0.8 are taking the value of −1 and colored in light blue, and PI values less than 0.5 are taking the value of −2 and colored in dark blue.

The sensitivity of the regulation network to molecule perturbations is investigated in order to assess the nodes’ significant effect on the system’s dynamics. The heatmap in (Figure 5) illustrates the effect of knocking out each node by 20% in the system on the other nodes. This was due to using the knockout matrix function, creating a heatmap function, and adjusting the sensitivity to 20% in the SPIDDOR package. The numerical range in the legend represents the ratio of the steady state of the regulatory network nodes between the perturbed and normal conditions (Perturbation Index (PI)). A value of 2 implies PIs larger than 2, a value of 1 implies PIs between 1.25 and 2, a value of 0 implies PIs close to 1, a value of −1 implies PIs between 0.8 and 0.5, and a value of −2 implies PIs less than 0.5. The heatmap has 30 rows, which corresponds to the number of proteins in the regulatory network. In general, knocking out each node in the network had little impact on the remaining nodes. In this scenario, the PIs are set to 0 and colored gray. The lower activation and inhibition of the nodes, represented by PI values of −1 and −2 and colored in light blue and dark blue, respectively, observed as a result of the perturbation is significantly greater than the higher activation of the nodes, represented by PI values of 1 and 2 and colored in light orange and dark orange, respectively. Clustering of perturbations was performed utilizing perturbed nodes that had a comparable effect on the network’s nodes.

For β-catenin, downstream of the Wnt pathway and the important molecule in maintaining cell proliferation, the patterns in the knockout heatmap indicated that maintaining the level of activity of Wnt and USP9X nearly to 100% of their normal activity is very important to retain the activity of β-catenin. GSK3β does not display a significant effect on β-catenin activity unless it is in combination with an imbalance of activity of Wnt or USP9X. For RPS6 and eIF4E, downstream of the mTOR pathway and the important molecules in maintaining the cell proliferation and survival, the patterns in the heatmap revealed that RPS6KA6/RSK4, FGF5, IDS, and SUMF1 preserving activity at nearly 100% of their normal activity is essential to sustain the activity of RPS6 and eIF4E. Cadherin protein is bounded on its activity with β-catenin, FLAN, and ACTB. As a cell surface molecule, its main function is to sustain cell adhesion. The knockout heatmap shows that the loss of the protein activity is in conjugation with the loss of FLAN, ACTB, and β-catenin. Similar to Cadherin, SMAD protein loss of function was observed only in the loss of function of FLNA or ACTB (Figure 5).

## 4. Discussion

Autism spectrum disorder (ASD) is a heterogeneous disorder that manifests itself through a wide variety of symptoms in different patients. Until now, a diverse array of genetic variants in various genes has been discovered. Precision medicine and annotation of the genetic mutations involved in the etiology of the disease are required to improve understanding of this complex disease. The ASD patients discussed in this study have genetic variants in multiple genes that have been confirmed to cause the disease in a study from [22]. Although the genes causing the disease were distinct, they shared their effects on the two most prevalent signaling pathways (mTOR and Wnt).

### 4.1. Genetic Variants Annotations

Patient 1 carries three genetic variants affecting three distinct disease-causing proteins: INTS6L/DDX26B, USP9X, and RPS6KA6/RSK4. According to the study, the genetic variant discovered in INTS6L/DDX26B is a novel mutation that has never been reported previously. Patient 2 has two genetic variants in FLNA and FGF5. The last two patients have a genetic variant in IDS and SUMF1, respectively. The role of these proteins in biological pathways and the specific range of amino acids on their polypeptide sequence that interact with other proteins were discovered through careful manual curation of these genetic variants in protein databases and a review of previous studies from PubMed about these proteins and their paralogs, families, and domains. Subsequently, the proteins carrying genetic variations have been incorporated into biological pathways to research the effect of the proteins’ dysfunction on those pathways and cellular function, thus explaining the appearance of the phenotype. The findings further interpret these proteins’ roles in the Wnt and mTOR pathways. Additionally, the two pathways have previously been implicated in the etiology of ASD [64,65]. After careful reviews, the underlying causes of ASD have been uncovered, providing valuable insights for the development of effective treatments.

Patient 1, INTS6L, lacked sufficient prior research on its structure or function to provide additional information about its disease-causing role in comparison to its paralog INTS6. Alignment of the sequences of these proteins revealed a high degree of identity, nearly 63%. By utilizing paralog studies, we were able to gain a better understanding of INTS6L’s functional effect in controlling the Wnt pathway [31,35]. USP9X is well-known for its ability to deubiquitinate and stabilize other proteins. Patient 1 has a mutation in USP9X’s deubiquitinating catalytic domain, which inhibits the protein’s interaction with β-catenin, a downstream effector of the Wnt signaling pathway, and RAPTOR, the activator of the mTOR signaling pathway [36,37]. RPS6KA6/RSK4 is highly expressed throughout the brain [39]. RSK enzymes activate the mTOR pathway by inhibiting TSC2 or activating RAPTOR via post-translational modification and by inhibiting GSK3β to accumulate β-catenin [41,43,44].

**Figure 6:**
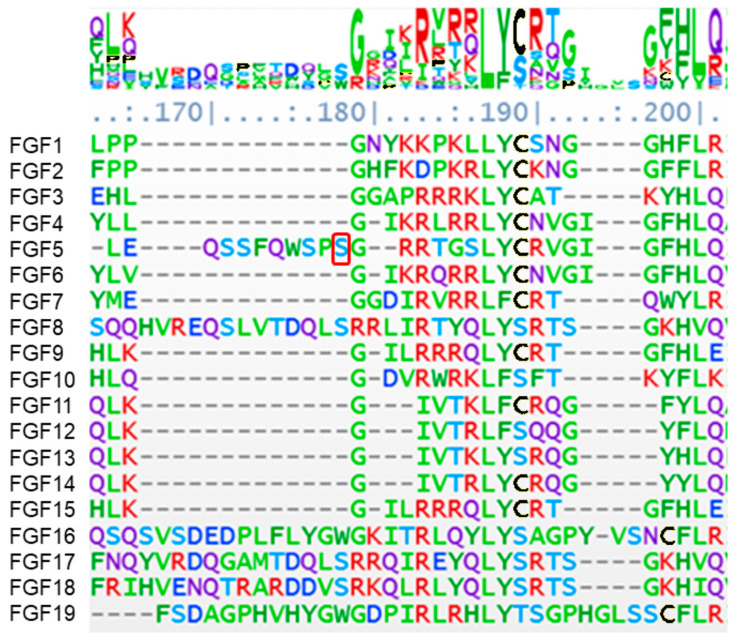
Multiple sequence alignment of the residues around beta strands 1 and 2 in the ligand FGF1-19 identified in [59] using NCBI COBALT and visualized with alignment viewer. The colored symbols are to ease comparison of same amino acid residue down the columns (yellow was recolored to black using onlinepngtools.com—accessed on 28 June 2024). The mutated residue (red box) in FGF5 is adjacent to the conserved domain. All sequences come from human cells, except FGF15, which is from mouse brain cells.

Patient 2 carries a mutation in FGF5. This ligand competes with FGFR1 for binding and activating the PI3K/Akt, RAS/MAPKs, and PLC/DAG pathways, which are involved in gene expression regulation and cell survival in the majority of cell types [66]. Previous studies using a structure-based multiple sequence alignment of the FGF family of proteins aligned the beta strands [59]. We have taken the sequences for human FGF1-19 from Uniprot (www.uniprot.com, accessed on 20 May 2024) and used the NCBI COBALT multiple alignment tool (https://www.ncbi.nlm.nih.gov/tools/cobalt/re_cobalt.cgi accessed on 20 May 2024) with the default settings visualized with AlignmentViewer.org (accessed on 20 May 2024) (Figure 6). The complete alignment is shown in Supplemental Figure S1. According to the protein structure in PDB, the missense mutation in FGF5 (p:S84L) in this ASD patient is located in the N-terminal alpha-helix prior to beta-sheet 1. This residue, located at position 178 in alignment in Figure 6, immediately precedes the highly conserved region from residue 179–192. It is the residues immediately preceding the highly conserved Y92 residue (188 in the alignment in Figure 6) that bind to the FGFR1 receptor in beta sheet 1 [67]. Previously, this regions was found to participate in competitive binding of the ligand to FGFR1 [68]. The proximity of the missense mutation suggests the importance of N-terminal alpha-helix, prior to beta sheet 1, in the ligand-receptor binding and gives the impression that the missense mutation in FGF5 is causing the ASD phenotype appearance because of the ligand’s inability to bind to its receptor.

Patient 2 also carries an FLNA mutation. This protein’s main functional role as an actin-binding protein is critical for the structure and shape of the cell membrane. Furthermore, FLNA interacts with other proteins involved in different cellular processes. The protein’s N-terminal region contains an actin-binding domain, and the C-terminal region contains 24 immunoglobulin (Ig)-like repeats [69]. According to the protein profile in the UniProt database, the missense mutation in FLNA (p:V2630A) in this ASD patient is located in repeat 22. Unfortunately, repeat 22 lacks sufficient research, but what has been found is that the proteins that interact with FLNA at repeat 22 are SMAD2 and β-arrestin [49,70,71]. Specifically, residues 2363 to 2647, which represent the C-terminal of repeat 22, were within the range of holding the interaction between FLNA and β-arrestin and within the range of amino acids near the FLNA missense mutation. The exact amino acid range for the interaction between these proteins is from 2386 to 2420. This interaction leads to activating ERK. Another two MAP2K proteins are interacting with FLNA in repeat 22. The residues that hold the interaction between FLNA and MEK1 are from amino acids 2282 to 2647, and the residues that hold the interaction between FLNA and MKK4 are from amino acids 2282 to 2454. The missense mutation in FLNA in this ASD patient is in both previous ranges [53]. What was interesting in the study of [53] that they made quintuple alanine-scanning substitutions in FLNA from the amino acid 2386 to 2426 to discover what are the amino acids in this range are the residues responsible for FLNA interacting with β-arrestin. The missense mutation in FLNA in patient 2 is the substitution of valine (V) to alanine (A), and this substitution causes the disease. However, when the polypeptide sequence of FLNA was reviewed in the UniProt database, position 2630 was alanine (A) and not valine (V). Our insights are that valine in this patient was a polymorphism, but what is interesting is that when valine is substituted for its original amino acid, alanine, it causes the disease. FLNA residues ranging from 2163 to 2517 were efficient in binding SMAD5 in vitro and SMAD1,2,4,5,6 proteins in vivo. FLNA was found to be an SMAD-binding protein that translocates the later protein to the nucleus and initiates the transcription of its targeted genes [51].

Patient 3 has a missense mutation in IDS (p:D175E), whereas patient 4 has a missense mutation in SUMF1 (p:Q237R). These two proteins are related. IDS is involved in the development of mucopolysaccharidosis type 2, a metabolic disorder known as Hunter syndrome that affects the face, respiratory system, heart, and central nervous system [28]. IDS is a lysosomal sulfatase enzyme that plays a critical role in protein metabolism, specifically in the degradation of large carbohydrates known as glycosaminoglycans from its substrates dermatan sulfate and heparan sulfate [54]. Heparan sulfate binds to FGF ligands and is a required molecule for activating the signal transduction pathways activated by the ligand binding to its receptors FGFRs [55]. However, excessive accumulation of heparan sulfate suppresses the FGF:FGFR interaction and alters the neural circuitry of the postnatal cortex, resulting in the ASD phenotype and ADHD [72]. It has been stated that the mutation of IDS causing excessive accumulation of heparan sulfate would impair the FGF-FGFR binding [73,74].

The majority of sulfatases contain an active site, denoted by a formyl glycine residue, which is critical for catalyzing the ester group formation. The active site motif of sulfatase enzymes is (C/S)XPXRXXX(S/T)G) [75], which we observed at position C84 of the IDS polypeptide sequence. It was mentioned that C171 and C184 in the IDS amino acid sequence form a disulfide bond between two short beta-strands, which is close to the catalytic site of the enzyme [76]. The IDS missense mutation D175E in patient 3 lies within the range of the disulfide bond formed between C171 and C184, indicating that the genetic variant distorted the catalytic site and prevented the enzyme from binding to its substrate.

SUMF1 acts as a cofactor for the IDS enzyme, enhancing its activity [56]. A mutation in SUMF1 results in a deficiency of sulfatase enzymes, as this molecule is required for the post-translational modification of these enzymes and is required for the modification of cysteine residue to formyl glycine [57]. In the original SUMF1 peptide sequence, position 237 is G, not Q, as it is in patient 4. The G237R missense mutation in SUMF1 has been implicated in the pathogenesis of lysosomal storage disease (LSD) in an analysis of the association between a potentially pathogenic variant in 42 LSD and cancer, determining which patients with LSD are at an increased risk of cancer [77]. Another study discovered an R236X mutation in SUMF1, resulting in the loss of 139 amino acids thought to be critical for protein stability and function, as well as the development of non-neurological symptoms [25]. The cystine residue at position 235, two amino acids prior to patient 4 mutation, forms a disulfide bond with the cystine residue at position 346. This disulfide bond aids in the stabilization of the protein’s core structure. Moreover, a mutation at position 234 would result in the loss of one hydrogen bond, impairing the core structure’s stability [78]. These findings suggest that a mutation in amino acid position 237 would impair the function and structure of the cofactor, resulting in the appearance of an ASD-like phenotype.

The first step in establishing that these mutations were responsible for the disease was manual curation and demonstration of the disruption in protein function caused by the genetic mutations. The second step should be to determine the extent of the damage caused by the genetic mutations and the effect on cell function. While Boolean network modeling is not widely used, this study demonstrated that the results from the developed tool (SPIDDOR) for visualizing network dynamics were helpful in elucidating the disease cause. The observation of the effect of perturbations on the activity of nodes in the network corroborates the role of these mutations in ASD pathology. Two suggestions were made regarding the effect of these genetic variants on protein function: partial or complete loss of function. These suggestions were chosen to explicate the effect of genetic mutations on the activity level of key proteins involved in cell proliferation and survival. Our findings indicated a distinction between the four patients.

### 4.2. Genetic Alterations’ Impact on Cellular Function

In patient 1, the Wnt pathway was overactive, as shown in Figure 4. This is because the activity of β-catenin was increased. Three proteins in patient 1 are altered: INTS6L, USP9X, and RPS6KA6/RSK4. The competition between the three proteins for activating or inhibiting the regulatory network’s components would have an influence on the molecules’ degree of activity. For instance, loss of function of INTS6L alone would result in an overactivation of the Wnt and mTOR pathways. The suppression of GSK3β and its influence on TSC1/2 activation [79] led to the overactivation of the mTOR pathway. RPS6KA6/RSK4 reduces TSC1/2 activity. However, the lack of function of this protein, including INTS6L, results in the loss of TSC1/2 function. GSK3β inhibition as a result of the overactivation of the Wnt pathway caused by the INTS6L mutation and the fact that Akt suppresses and replaces RPS6KA6/RSK4 in reducing TSC1/2 activity would account for TSC1/2 loss of function. Even if TSC1/2 is present in trace amounts, Akt inhibits its action, and GSK3β is already inhibited in its activation. This would result in an over-activation of the mTOR pathway, but in patient 1, this pathway is suppressed in Figure 4.

The mutation in the third protein, USP9X, in patient 1, in addition to the mutation in RPS6KA6/RSK4, is leading to the inhibition of the mTOR pathway. RAPTOR is a critical activator of the mTOR pathway. Rapamycin inhibits the mTOR pathway by preventing RAPTOR and MTORC1 from interacting [80]. USP9X protects the mTOR pathway in human brain progenitor cells by stabilizing RAPTOR, preventing its degradation via the proteosome degradation pathway [36]. RSK enzymes phosphorylate RAPTOR in three serin sites and activate the mTOR pathway through the RAS/MAPK pathway and independently from the PI3K/Akt pathway [41]. Together, the loss of function of USP9X and RPS6KA6/RSK4 inhibits RAPTOR, which would account for the suppression of the mTOR pathway in patient 1 in Figure 4.

Previous research indicated that increased levels of β-catenin are a factor influencing ASD-like defects and contributing to disorder pathogenesis [81,82,83,84]. Also, previous findings implied that the mTOR pathway is downregulated in individuals with ASD. This includes the proteins MTORC1, RPS6, and eIF4E [65,85,86]. Cadherin protein activity was increased in patient 1 in correlation with increased β-catenin levels. Because the Boolean definition of Cadherin is dependent on the activity of β-catenin and F-actin, any change in the activity of these two proteins will impact Cadherin activity. This is a disadvantage of Boolean models, as they do not convey relative regulation due to the use of 0 and 1. Meanwhile, disruption of the Cadherin protein has been implicated as a risk factor for ASD. They are required for the growth and maturation of neurons and brain networks [65,87,88].

In patient 2, the Wnt pathway was slightly increased in Figure 4. The patient has a mutation in two proteins: FLNA and FGF5. Again, the competition between the two proteins for activating or inhibiting the regulatory network’s molecules would affect the extent to which these molecules are active or inactive. FLNA is an actin-binding protein that helps shape the cell’s cytoskeleton. This protein contains an N-terminal domain that interacts with actin, and a C-terminal domain contains 24 Ig-like repeats that interact with other proteins [49]. Apart from the FLNA genetic variations that result in the protein gaining or losing function [89], this patient has a mutation in FLNA at repeat 22. Although there is a scarcity of studies on repeat 22 and its binding protein, evidence from [53] demonstrates that FLNA binds to β-arrestin in repeat 22, and the range of this patient genetic variant is located in this binding site. Binding the two proteins activates ERK1/2 via MEK, and colocalization of the three proteins to membrane ruffles is required for cell migration [53]. Cadherin protein, which was shown to be deficient in patient 2 due to the FLNA mutation, is localized in membrane ruffles together with its binding proteins to promote cell motility by promoting actin polymerization in lamellipodia [90]. Another protein binding to FLNA in repeat 22 is SMAD. In patient 2, the SMAD protein was observed to be inhibited solely due to an FLNA protein mutation. SMAD proteins have been implicated in TGF-β signaling. When active, the TGF-β receptor phosphorylates SMAD proteins, allowing them to translocate to the nucleus and induce transcription of specific genes [91]. SMAD proteins translocation to the nucleus combines with binding to FLNA. A defect in TGF-β signaling was detected in association with FLNA deficiency, as well as the inability of the SMAD protein binding to FLNA, which is required for translocation to the nucleus and transcription initiation [51]. Additionally, a decrease in the TGF-β pathway was observed in ASD patients [92], with SMAD proteins being a risk factor in ASD patients’ gene enrichment study [93]. In patient 2, the FGF5 mutation prevents the ligand from binding to its receptor. As a result, inhibitions of signaling pathways would be dependent on receptor activity. As a result, the mTOR signaling pathway should be inhibited. Because molecules in the regulatory network are inhibited and activated by various factors within the cell, β-arrestin activates PI3K, which activates Akt and inhibits TSC1/2. β-arrestin also activates RAF, which is upstream of ERK1/2, which activates RPS6KA6/RSK4 and inhibits TSC1/2. RPS6KA6/RSK4 is likewise activated downstream of PI3K by PDK. Again, the competing molecules within the cell for inhibition or activation would result in a drop in the mTOR pathway, although not to the amount seen in patient 1.

Patients 3 and 4 can be considered as Multiple Sulfatases Deficiency (MSD) patients as a mutation in IDS and SUMF1 are related to this disease and exhibit the same pattern of protein activity in Figure 4. Only the mTOR pathway was affected and decreased. This would also corroborate the previous article’s assertion that ASD patients have abnormal mTOR activation [94]. Recognizing the activity level of the major proteins involved in cell function and shape maintenance would aid in tailoring the treatment and dosage required to reverse the effect of the genetic mutations in all patients. The results of Boolean analysis assisted in highlighting the activity levels of β-catenin, MTORC1, RPS6, eIF4E, cadherin, and SMAD. To validate our study’s findings, we can compare these proteins’ activity levels to their expression levels in the gene expression profile results from ASD patients’ medical files. Advising on the treatment is a difficult task due to the fact that the proteins in both pathways are regulatory factors for one another.

### 4.3. Possible Alternative Therapies

Aripiprazole and Risperidone are atypical antipsychotic drugs that have been licensed by the FDA for the treatment of ASD. Their method of action is based on antagonizing the Dopamine receptor type 2 (D2R) and the serotonin 2A receptor (5-HT2A)/ β-arrestin2 pathway, respectively [95,96]. Aripiprazole has a greater affinity for partly agonizing/antagonizing D2R receptors, depending on the dopamine neurotransmitter density state [97,98]. Risperidone has a greater affinity for inhibiting the 5-HT2A receptor than the D2R receptor [99]. D2R or 5-HT2A activation results in the recruitment of β-arrestin 2 to the receptor and an increase in Akt and protein phosphatase 2 (PP2A) binding, inhibiting Akt activity and activating GSK3β, an Akt downstream effector [96,99,100,101,102]. In the case of patients 1 and 2, activation of GSK3β would aid in regulating β-catenin overexpression but would inhibit the mTOR pathway, as GSK3β activates the TSC1/TSC2 complex, and the mTOR pathway already has a low activity. Synergy of D2R or 5-HT2A antagonists and agonists would be a better therapy option for controlling GSK3β activation/inhibition via the Akt enzyme [103,104,105,106]. Furthermore, a recent study found that the bioagent “NV-5138” has a high selectivity for activating mTORC1 in the brain via sestrin2 binding. The study demonstrated a favorable outcome in terms of mTORC1 inhibition in neuropsychiatric diseases. Navitor Pharmaceuticals is developing it [107]. Combining this bioagent with a specific activator of GSK3β may be a possibility.

Overall, the proposed treatment is a selective GSK3β activator to suppress β-catenin overexpression in combination with a selective mTORC1 activator to activate RPS6 and eIF4E expression. Since the only pathway affected is the mTOR pathway, could rapamycin be considered as a treatment option for patients 3 and 4? Research has found that because autophagy was observed throughout the whole CNS in IDS knockout mice, rapamycin was not a good option in treatment, and instead, the researchers suggested chloroquine to manage the autophagy [108].

Patients 3 and 4 are considered as Multiple Sulfatases Deficiency (MSD) patients as mutations in IDS and SUMF1 are related to this disease [109,110]. The mutation in either IDS or SUMF1 causes an alteration in heparan sulfate metabolism, which would cause autistic-like symptoms [29]. The researchers found that autistic-like behavior is due to high Dopamine receptor type 1 (D1R) activity in the striatum. They managed these behaviors with an introduction of a D1R inhibitor, and they suggested considering the pathological changes in any Heparan Sulfate-mediated signaling when it comes to therapeutic treatment.

There has been a substantial surge in the number of genes linked to ASD. It is essential to examine the genes responsible for ASD in every case. Additionally, several molecular pathways that contribute to the development of ASD have been identified. The current direction is to target the discovered genes and pathways for innovative therapeutic methods [111]. It is essential to define and analyze the molecular pathways in order to understand the behavior of molecules that affect the emergence of the phenotype. Boolean network models, a form of graphical qualitative models, have been employed in the study of biological regulation networks [112,113,114]. These forms of network analysis are capable of understanding the dynamic behavior demonstrated by the network components [15,20]. In this study, a Boolean Network analysis offered by the SPIDDOR package in R was used to analyze seven proteins related to four autism patients. Boolean algorithms illustrate basic patterns of activation and inhibition among the nodes. The SPIDDOR software enhances the precision of describing biological reactions by introducing modulation functions that temporarily delay or enhance the activation or inhibition states of nodes impacted by other nodes’ activity. It also implements mutation-like status for proteins with genetic variants to assess their impact on the network and understand the underlying cause behind phenotype appearance. The package includes sensitivity analysis and clustering techniques to identify nodes with similar impact on the network when perturbed. These advantages are not all present in other developed tools for Boolean network analysis, such as BoolNet [115], BooleanNet [116], BoolSi [117], and ViSiBooL [118]. It is apparent that these tools lack the modulation functions and sensitivity analysis provided by the SPIDDOR package. The advantage of this tool was that it allowed for the changing of the activity rate of some proteins in the network. These modifications mimic the effect of genetic mutations on protein function in order to assist in determining the impact of genetic mutations of certain proteins on the activity of other proteins in the network. Furthermore, the simulation of the network with some proteins’ activity modified highlighted the defects in the biological pathways and identified specific targets in the network in order to treat these patients by reversing the defect that occurred in the biological pathways and improving the developed phenotype. Additional examination of the expression of these biological pathways’ molecules in the gene expression profile of these patients is necessary. In general, it is advisable to utilize Boolean network modeling due to the absence of the expression profile of these patients and the requisite quantities for quantifying the extent of the impact of the mutant genes on the appearance of the disease. Additionally, the utilization of Boolean network modeling functions as a valuable tool for this study and has the potential to be applied to other complex biological networks in the future.

### 4.4. Model Limitations

To our knowledge this is the only model constructed for this set of genes to describe autism spectral disorder. The current model contains one loop; however, the interactions in the loop are bi-directional. There are no directed cyclic loops that would describe feedback (positive or negative). To our knowledge, feedback loops have not been identified for the system modeled and are thus not included in the model. The existence of feedback loops could impact the behavior of the model. For example, a positive feedback loop has been proposed in the PI3K-Akt-mTORC1-SREBP signaling cascade that activates Akt signaling in human melanoma cells expressing GD3, which can account for unchecked cell growth [119]. Further exploration in this regard is possible in future work.

## 5. Conclusions

The Boolean analysis technique used in this work provides insight into the phenotype origin, pathophysiology, and therapy choices for autistic patients. Although ASD is a heterogeneous disorder, the study [22] identifies the genetic variants responsible for the disorder. This simplifies annotating these variants and incorporating them into biological pathways to reveal the cause, which includes a de novo variant in INTS6L. The Boolean network analysis method aids in identifying the most critical proteins (β-catenin, RPS6, eIF4E, Cadherin, and SMAD) that are influenced by genetic variations; nevertheless, further examination of these proteins’ expression in the autistic patients’ files is required. The intriguing part is that the major proteins necessary for cell integrity (β-catenin, RPS6, eIF4E, Cadherin, and SMAD) have previously been implicated in the etiology of ASD.

The manual annotation provided clarification regarding the convergence of these proteins within the mTOR and Wnt pathways. The protein–protein interaction network reviewed and analyzed in this study is a simple directed graph with no feedback loops included. For this reason, the BooTablelean network analysis method emerged as the most appropriate graphical analytic approach to be used, and it aids in revealing the hidden realities underneath the phenotype appearance. For future work, employing more advanced network analysis techniques such as cyclic directed graphical models, dependency network models, or any type of graphical statistical probabilistic model that allows for cyclic direction in feedback loops in regulatory networks should be considered. This consideration should be given in light of the accessibility of gene expression data and microarray data. This will make the results more informative in terms of protein–protein interaction networks and cell physiology.

## Figures and Tables

**Table 1:** The number of patients and the genetic variants considered in this study and collected from the study of [22]. DC: disease-causing, PD: Possibly Damaging, B: Benign, D: Damaging. Mutation Taster and PolyPhen are two online tools for predicting the genetic variants effect.

	Gender	Clinical Demographic Information	Protein Name	Variant Position	Effect of the Variant	
					Mutation Taster	PolyPhen
Patient 1	F	Language delay and regression	DDX26B /INTS6L	p:E435V	DC	PD/0.843
			USP9X	p:Y1268C	DC	B/0.007
			RPS6KA6 /RSK4	p:Q512R	DC	B/0.195
Patient 2	M	NR	FGF5	p:S84L	DC	D/1.0
			FLNA	p:Y2360A	DC	D/0.971
Patient 3	M	Language delay	IDS	p:D175E	DC	PD/0.94
Patient 4	M	Language delay	SUMF1	p:Q237R	DC	D/1.0

**Table 2:** The values and the colors assigned to the ranges of the perturbation index (PI) at 20% sensitivity.

Low	High	Value	Color
≤0.5		−2	Dark Blue
0.5	0.8	−1	Light Blue
0.8	1.25	0	Gray
1.25	2.0	1	Light Orange
≥2.0		2	Dark Orange

**Table 3:** An illustration of how the dynamic evolution simulation is performed.

**Running the dynamic evolution function in the physiological state of the protein**													
Steps	1	2	3	4	5	6	7	8	9	10	---	99	100
INTS6L	1	1	0	1	0	1	0	1	0	1	---	0	1
**Running the dynamic evolution function when the mutation-like effect was delaying the protein activity with a 50% rate**													
INTS6L	0	0	1	0	0	0	0	1	0	1	---	0	1

**Table 4:** Number of states that the Boolean network oscillates in while running the simulation to compute the steady states of the nodes in the network at the normal condition and the perturbed conditions.

	Patient1 (INTS6L, USP9X, RPS6KA6)	Pateint2 (FLNA, FGF5)	Pateint3 (IDS)	Pteint4 (SUMF1)
Normal conditions of the network	321	321	321	321
50% activity of proteins with variant	103	158	185	102
0% activity of proteins with variant	1229	224	243	236

## Data Availability

All data generated or analyzed during this study are included in this published article and its supplementary information files. The number of patients and the genetic variants considered in this article were collected from the study of [22]. The PPI model and other code are shared at https://github.com/lnezamuldeen/PPI_creation_models (accessed 30 June 2024).

## Supplementary Materials

The following supporting information can be downloaded at https://www.mdpi.com/article/10.3390/biology13080606/s1. Figure S1. Full alignment of FGF proteins using NCBI COBALT.

## Appendix A

**Table A1:** Boolean functions describe the relations between the nodes in the network.

Node	Boolean Function	Explanation	Reference
INTS6L	INTS6L = ! (INTS6L & ANY_INTS6L[neg_modulator])	To check if INTS6L is activated in any of the iterations of the simulation and not affected by any of its negative modulator	[21]
WIF1	WIF1 = INTS6L &! (MOD_WIF1)	INTS6L is mediating the expression of WIF1 if WIF1 is activated	[34,35]
WNT	WNT = ! WIF1	WNT is inhibited by WIF1	[34,35]
BArrestin	BArrestin = ! (BArrestin & ANY_BArrestin[neg_modulator])	To check if BArrestin is activated in any of the iterations of the simulation and not affected by any of its negative modulator	[53]
Dsh	Dsh = WNT & BArrestin	BArrestin binds to disheveled protein upon Wnt pathway activation	[120]
SUMF1	SUMF1= ! (SUMF1& ANY_ SUMF1[neg_modulator])	To check if SUMF1is activated in any of the iterations of the simulation and not affected by any of its negative modulator	[25]
IDS	IDS = SUMF1	*SUMF1* is a cofactor to enhance the activity of IDS	[57]
ACTB	ACTB = BArrestin &! (MOD_ACTB)	BArrestin mediates the activation of cofilin, severing existing filaments to release free actin monomers to assemble into a new filament	[121]
FLNA	FLNA = ACTB &! (MOD_FLAN)	FLNA binds ACTB to form a network of filaments that acts as the cell cytoskeleton. Also, it is involved in cell adhesion, migration, and determination of shape.	[62,122,123]
F_actin	F_actin = ACTB & FLNA	Filament protein that is assembled from actin binding to filamin A	[62,122,123]
FGF5	FGF5 = IDS & !(MOD_FGF5)	Heparan sulfate, degraded by IDS, is a specific and central component in *FGF/FGFR* binding Mutation in IDS causes accumulation of heparan sulfate and leads to impaired FGF-FGFR binding	[55,73,74]
FGFR1	FGFR1 = FGF5	FGF5 binds specifically to FGFR1	[124,125]
PI3K	PI3K = BArrestin|FGFR1	PI3K activation induced either by BArrestin or FGFR1 activation	[66,126]
PDK	PDK = PI3K	PDK activated through PI3K	[66]
Akt	Akt = PDK	Akt activated by PDK	[66]
RAS	RAS = FGFR1	RAS activated by FGFR1 activation	[66]
RAF	RAF = RAS|BArrestin	RAF activation induced either by BArrestin or RAS activation	[66,127]
MEK	MEK = RAF|(BArrestin & FLNA)	MEK activated either by RAF or BArrestin binding to FLNA	[53,66]
ERK1/2	ERK1/2 = MEK|(BArrestin & FLNA)	ERK1/2 activated either by RAF or BArrestin binding to FLNA	[53,66]
RPS6KA6	RPS6KA6 = ERK12	RPS6KA6 is activated by ERK12	[128]
GSK3B	GSK3B = ! (WNT|RPS6KA6 | Akt)	GSK3B is inhibited by the activation of WNT OR RPS6KA6 OR Akt	[44]
USP9X	USP9X = ! WNT|! (MOD_USP9X)	In the absence of WNT OR to check if USP9X is activated in any of the iterations of the simulation and not affected by any of its negative modulators	[21,37]
BCATENIN	BCATENIN= (GSK3B & USP9X)|(WNT &! GSK3B)	In the absence of WNT, where GSK3B is active and ubiquitinates B-CATENIN and USP9X deubiquitinates B-CATENIN and saves it from proteasomal degradation OR in the presence of WNT.	[44]
Cadherin	Cadherin = F_actin & BCATENIN	Alpha Catenin links BCATENIN to Actin and promotes BCATENIN binding to Cadherin for cell adhesion processes.	[62]
RAPTOR	RAPTOR = USP9X|RPS6KA6	RAPTOR is activated by USP9X OR RPS6KA6	[36,41]
TSC12	TSC12 = GSK3B &! (RPS6KA6|Akt)	TSC12 is activated by GSK3B and inhibited by either RPS6KA6 OR Akt	[129,130]
MTORC1	MTORC1 = RAPTOR &! TSC12	MTORC1 is activated by RAPTOR and inhibited by TSC12	[36,129,130]
RPS6	RPS6 = MTORC1 | RPS6KA6	RPS6 is activated by MTORC1	[36]
eIF4E	eIF4E = MTORC1 | RPS6KA6	eIF4E is activated by MTORC1	[36]
SMAD	SMAD = FLNA	SMAD binds to FLNA to translocate to the nucleus and transcribe targeted genes	[51]
