# Supplementary material for: Boolean Modeling of Biological Network Applied to Protein–Protein Interaction Network of Autism Patients
Source: Biology (Basel). 2024 Aug 10;13(8):606. doi: 10.3390/biology13080606 (PMC11352122; doi:10.3390/biology13080606)
Supplement: Supplementary file 1 [file biology-13-00606-s001.zip › biology-3068107-supplementary.pdf]

```
FGF1   1      ---------------------------   ----------------MAEGEITTFTA   LTEKFN----LPP      20
FGF2   1  [83]RGRALPGGRLGGRGRGRAPerVGGRGR[7]RAAPAARGSRPGPAGTMAAGSITTLPA   LPEDGGSG-AFPP     156
FGF3   1      ---------------------------[6]LLLSLLEPGWP------AAGPGARLRR   DAGGRGGV-YEHL      39
FGF4   1  [11]LPAVLLALLAPWAGRGGAAapTAPNGT   LEAELERRWESLVALSLARLPVAAQPK   EAAVQSGAgDYLL      78
FGF5   1  [10]FSHLILSAWAHGEKRLAPKgqPGPAAT   DRNPRGSSSRQSSSSAMSSSSASSSPA[1]SLGSQGSG---LE[ 9]  84
FGF6   1  [22]WALVFLGILVGMVVPSPAG--TRANNT   LLDS--RGWGTL--LSRSRAGLAGEIA   GVNWESG---YLV      80
FGF7   1  [17]--CFHIICLVGTISLACND--------   MTPEQM---ATN---VNCSS------P   ERHTRSY--DYME      60
FGF8   1      ---------------------------[5]SALSCLL-LHLLVLCLQAQEGPGRGPA[9]RAGREPQG-VSQQ[13]  65
FGF9   1  [15]------AVPFGNVPVLPVDspVLLSDH   LGQS----------------EAGGLPR   GPAVTDL--DHLK      58
FGF10  1  [20]CCCFLLLFLVSSVPVTCQA--------   LGQDMVSPEATN---SSSSSFSSPSSA   GRHVRSY--NHLQ      74
FGF11  1      MAA-LASSLIRQKREVREPggSRPVSA[6]RGT-KSLCQKQLLILLSKVRLCGGRPA   RPDRGPEP--QLK      69
FGF12  1      MAAAIASSLIRQKRQARESnsDRVSAS[6]SKDGRSLCERHVLGVFSKVRFCSGRKR   PVRRRPEP--QLK      71
FGF13  1      MAAAIASSLIRQKRQARER--EKSNAC[6]SKG-KTSCDKNKLNVFSRVKLFGSKKR   R-RRRPEP--QLK      67
FGF14  1      MAAAIASGLIRQKRQAREQhwDRPSAS[6]SKN-RGLCNGNLVDIFSKVRIFGLKKR   RLRRQ-DP--QLK      69
FGF15  1  [12]WDLHGFSSSLGNVPL--ADspGFLNER   LGQI----------------E-GKLQR   G-SPTDF--AHLK      57
FGF16  1      ---------------------------   ------MARKWNGRAVARALVLATLWL   AVSGRPLA-QQSQ[13]  46
FGF17  1      ---------------------------[5]LLPNLTLCLQLLILCCQTQ--------   -GENHPSP-NFNQ[13]  48
FGF18  1      ---------------------------[5]SACTC-LCLHFLLLCFQVQVLVA----   ----EENV-DFRI[13]  48
FGF19  1      ---------------------------   ------MR---SGCVVVHVWILAGLWL   AVAGRPLA-----[12]  38
```

```
FGF1   21   GnYKKPKLLYCSNG   GHFLRILPDGTVDGTRDRSDQHIQLQLSAES-VGEVYIKSTETGQYLAMDTDGLLYGSQ-TPN   95
FGF2   157  GhFKDPKRLYCKNG   GFFLRIHPDGRVDGVREKSDPHIKLQLQAEE-RGVVSIKGVCANRYLAMKEDGRLLASK-CVT  231
FGF3   40   GgAPRRRKLYCAT-   KYHLQLHPSGRVNGSLENS-AYSILEITAVE-VGIVAIRGLFSGRYLAMNKRGRLYASE-HYS  112
FGF4   79   G-IKRLRRLYCNVG[1]GFHLQALPDGRIGGAHAD-TRDSLLELSPVE-RGVVSIFGVASRFFVAMSSKGKLYGSP-FFT  152
FGF5   85   G--RRTGSLYCRVG[1]GFHLQIYPDGKVNGSHEA-NMLSVLEIFAVS-QGIVGIRGVFSNKFLAMSKKGKLHASA-KFT  157
FGF6   81   G-IKRQRRLYCNVG[1]GFHLQVLPDGRISGTHEE-NPYSLLEISTVE-RGVVSLFGVRSALFVAMNSKGRLYATP-SFQ  154
FGF7   61   GgDIRVRRLFCRT-   QWYLRIDKRGKVKGTQEMKNNYNIMEIRTVA-VGIVAIKGVESEFYLAMNKEGKLYAKK-ECN  134
FGF8   66   RrLIRTYQLYSRTS   GKHVQVLANKRINAMAEDGDPFAKLIVETDTfGSRVRVRGAETGLYICMNKKGKLIAKSnGKG  142
FGF9   59   G-ILRRRQLYCRT-   GFHLEIFPNGTIQGTRKDHSRFGILEFISIA-VGLVSIRGVDSGLYLGMNEKGELYGSE-KLT  131
FGF10  75   G-DVRWRKLFSFT-   KYFLKIEKNGKVSGTKKENCPYSILEITSVE-IGVVAVKAINSNYYLAMNKKGKLYGSK-EFN  147
FGF11  70   G---IVTKLFCRQG   -FYLQANPDGSIQGTPEDTSSFTHFNLIPVG-LRVVTIQSAKLGHYMAMNAEGLLYSSP-HFT  140
FGF12  72   G---IVTRLFSQQG   -YFLQMHPDGTIDGTKDENSDYTLFNLIPVG-LRVVAIQGVKASLYVAMNGEGYLYSSD-VFT  142
FGF13  68   G---IVTKLYSRQG   -YHLQLQADGTIDGTKDEDSTYTLFNLIPVG-LRVVAIQGVQTKLYLAMNSEGYLYTSE-LFT  138
FGF14  70   G---IVTRLYCRQG   -YYLQMHPDGALDGTKDDSTNSTLFNLIPVG-LRVVAIQGVKTGLYIAMNGEGYLYPSE-LFT  140
FGF15  58   G-ILRRRQLYCRT-   GFHLEIFPNGTVHGTRHDHSRFGILEFISLA-VGLISIRGVDSGLYLGMNERGELYGSK-KLT  130
FGF16  47   GkITRLQYLYSAGP[3]NCFLRIRSDGSVDCEEDQ-NERNLLEFRAVA-LKTIAIKDVSSVRYLCMSADGKIYGLIrYSE  124
FGF17  49   RrQIREYQLYSRTS   GKHVQV-TGRRISATAEDGNKFAKLIVETDTfGSRVRIKGAESEKYICMNKRGKLIGKPsGKS  124
FGF18  49   RkQLRLYQLYSRTS   GKHIQV-LGRRISARGEDGDKYAQLLVETDTfGSQVRIKGKETEFYLCMNRKGKLVGKPdGTS  124
FGF19  39   GdPIRLRHLYTSGP[4]SCFLRIRADGVVDCARGQ-SAHSLLEIKAVA-LRTVAIKGVHSVRYLCMGADGKMQGLLqYSE  117
```

```
FGF1   96  EECLFLERLEENHYNTYISKKH---     AEKNWFVGLKKNGSCKRGPR--THYGQKAILFLPLPVSSD----------- 155
FGF2   232 DECFFFERLESNNYNTYRSRKY---     T--SWYVALKRTGQYKLGSK--TGPGQKAILFLPMSAKS------------ 288
FGF3   113 AECEFVERIHELGYNTYASRLYRTV[11]AERLWYVSVNGKGRPRRGFK--TRRTQKSSLFLPRVLDHRD---HEMVRQL 194
FGF4   153 DECTFKEILLPNNYNAYESYKYPGM     -----FIALSKNGKTKKGNR--VSPTMKVTHFLPRL--------------- 206
FGF5   158 DDCKFRERFQENSYNTYASAIHRTE[ 1]TGREWYVALNKRGKAKRGCSprVKPQHISTHFLPRFKQSEQ---PELSFTV 231
FGF6   155 EECKFRETLLPNNYNAYESDLYQGT     -----YIALSKYGRVKRGSK--VSPIMTVTHFLPRI--------------- 208
FGF7   135 EDCNFKELILENHYNTYASAKWTHN     -GGEMFVALNQKGIPVRGKK--TKKEQKTAHFLPMAIT------------- 194
FGF8   143 KDCVFTEIVLENNYTALQNAKYE--     ---GWYMAFTRKGRPRKGSK--TRQHQREVHFMKRLPRGH----HTTEQSL 207
FGF9   132 QECVFREQFEENWYNTYSSNLYKHV[ 1]TGRRYYVALNKDGTPREGTR--TKRHQKFTHFLPRPVDPDK--vPELYKDI 204
FGF10  148 NDCKLKERIEENGYNTYASFNWQHN     -GRQMYVALNGKGAPRRGQK--TRRKNTSAHFLPMVVHS------------ 208
FGF11  141 AECRFKECVFENYYVLYASALYRQR[ 1]SGRAWYLGLDKEGQVMKGNR--VKKTKAAAHFLPKLLEVAM--yQEPSLHS 213
FGF12  143 PECKFKESVFENYYVIYSSTLYRQQ[ 1]SGRAWFLGLNKEGQIMKGNR--VKKTKPSSHFVPKPIEVCM--yREPSLHE 215
FGF13  139 PECKFKESVFENYYVTYSSMIYRQQ[ 1]SGRGWYLGLNKEGEIMKGNH--VKKNKPAAHFLPKPLKVAM--yKEPSLHD 211
FGF14  141 PECKFKESVFENYYVIYSSMLYRQQ[ 1]SGRAWFLGLNKEGQAMKGNR--VKKTKPAAHFLPKPLEVAM--yREPSLHD 213
FGF15  131 RECVFREQFEENWYNTYASTLYKHS[ 1]SERQYYVALNKDGSPREGYR--TKRHQKFTHFLPRPVDPSK--lPSMSRDL 203
FGF16  125 EDCTFREEMDCLGYNQYRSMKH---     -----HLHIIFIQAKPREQL---QD-QKPSNFIPVFHRSFF--eTGD---Q 183
FGF17  125 KDCVFTEIVLENNYTAFQNARHE--     ---GWFMAFTRQGRPRQASR--SRQNQREAHFIKRLYQGQLpfpNHAEKQK 193
FGF18  125 KECVFIEKVLENNYTALMSAKYS--     ---GWYVGFTKKGRPRKGPK--TRENQQDVHFMKRYPKGQ------PELQK 187
FGF19  118 EDCAFEEEIRPDGYNVYRSEKHR--     ----LPVSLSSAKQRQLYKN---RGFLPLSHFLPMLPMVPE--ePEDLRGH 182
```

```
FGF1       -------    ----    ---------
FGF2       -------    ----    ---------
FGF3   195 QSGLPRP[5]QPRR[4]QSPDNLEpS[16]  239
FGF4       -------    ----    ---------
FGF5   232 TVPEKKK[5]KPKI[4]PRKNTNSvK[ 8]  268
FGF6       -------    ----    ---------
FGF7       -------    ----    ---------
FGF8   208 RFEFLNY[2]FTRS[4]QRTWAPEpR      233
FGF9   205 LSQS---    ----    ---------    208
FGF10      -------    ----    ---------
FGF11  214 VPEASPS[4]P---    ---------    225
FGF12  216 IGEKQGR    -SRK[4]PTMNGGKvV[ 5]  243
FGF13  212 LTEFSRS[4]PTKS[4]GVLNGGKsM[ 6]  245
FGF14  214 VGETVPK[4]PSKS[4]AIMNGGKpV[ 6]  247
FGF15  204 FHYR---    ----    ---------    207
FGF16  184 LRSKMFS[3]ESDS[4]RMVEDVDhL[ 8]  218
FGF17  194 QFEFVGS[2]TRRT    KRTRRPQpL[ 1]  216
FGF18  188 PFKYTTV    TKRS    RRIRPTHpA      207
FGF19  183 LESDMFS[3]ETDS[4]GLVTGLE-A[ 8]  216
```

Figure S1. Full Alignment of FGF proteins using NCBI COBALT.
